# Crystal structure of 2-methyl-1*H*-imidazol-3-ium 3,5-di­carb­oxy­benzoate

**DOI:** 10.1107/S2056989023009209

**Published:** 2023-10-31

**Authors:** Sofiia Baletska, Simone Techert, Jose de Jesus Velazquez-Garcia

**Affiliations:** a School of Physics, V. N. Karazin Kharkiv National University, 4 Svobody Sq., Kharkiv 61022, Ukraine; b Deutsches Elektronen-Synchrotron DESY, Notkestr. 85, 22607 Hamburg, Germany; c Institut für Röntgenphysik, Georg-August-Universität Göttingen, Friedrich-Hund-Platz 1, Göttingen, 37077, Germany; Universidad Nacional Autónoma de México, México

**Keywords:** crystal structure, 2-methyl­imidazole, trimesic acid

## Abstract

The structure of the title salt, C_4_H_7_N_2_^+^·C_9_H_5_O_6_^−^ (**1**), is reported. The compound is built from a protonated 2-methyl­imidazole and a singly deprotonated trimesic acid. Detailed analysis of bond distances and angles for both ions reveals subtle differences compared with their neutral mol­ecule counterpart. Analysis of the crystal packing in compound **1** reveals the formation of undulating chains by the ions through hydrogen bonding. The chains stack along the *b* axis through π–π inter­actions and inter­connect with other chains in an out-of-phase arrangement along the *ac* plane through further hydrogen-bonding inter­actions.

## Abstract 2

The structure of a 2-methyl-1*H*-imidazol-3-ium trimesate compound was determined by single-crystal X-ray diffraction. The compound is composed of protonated 2-methyl­imidazole and singly deprotonated trimesic acid mol­ecules.

## 1. Chemical context

Trimesic acid, also known as 1,3,5-benzene­tri­carb­oxy­lic acid (Hbtc), and 2-methyl­imidazole (mIm) are two well-known organic compounds with significant applications in various industries. For example, mIm, a nitro­gen-containing heterocyclic organic compound, serves as a versatile chemical inter­mediate that is used extensively in the synthesis of pharmaceuticals, photographic and photothermographic chemicals, dyes and pigments, agricultural chemicals, and in rubber production (Hachuła *et al.*, 2010 ▸; Chan, 2004 ▸). On the other hand, Hbtc is a planar and highly symmetrical trifunctional compound, which finds use in coating materials, adhesives, plastics, and even in the pharmaceutical industry for drugs and gene carriers. Notably, some dendrimers based on Hbtc have been employed as biomolecular delivery systems (Salamończyk, 2011 ▸; Mat Yusuf *et al.*, 2017 ▸). Both Hbtc and mIm are also well-established ligands frequently employed in the synthesis of metal–organic frameworks (MOFs). For example, mIm is used in the synthesis of ZIF-8 (zeolitic imidazolate framework − 8; Park *et al.*, 2006 ▸), while Hbtc is employed in the production of HKUST-1 (Hong Kong University of Science and Technology − 1; Chui *et al.*, 1999 ▸). 
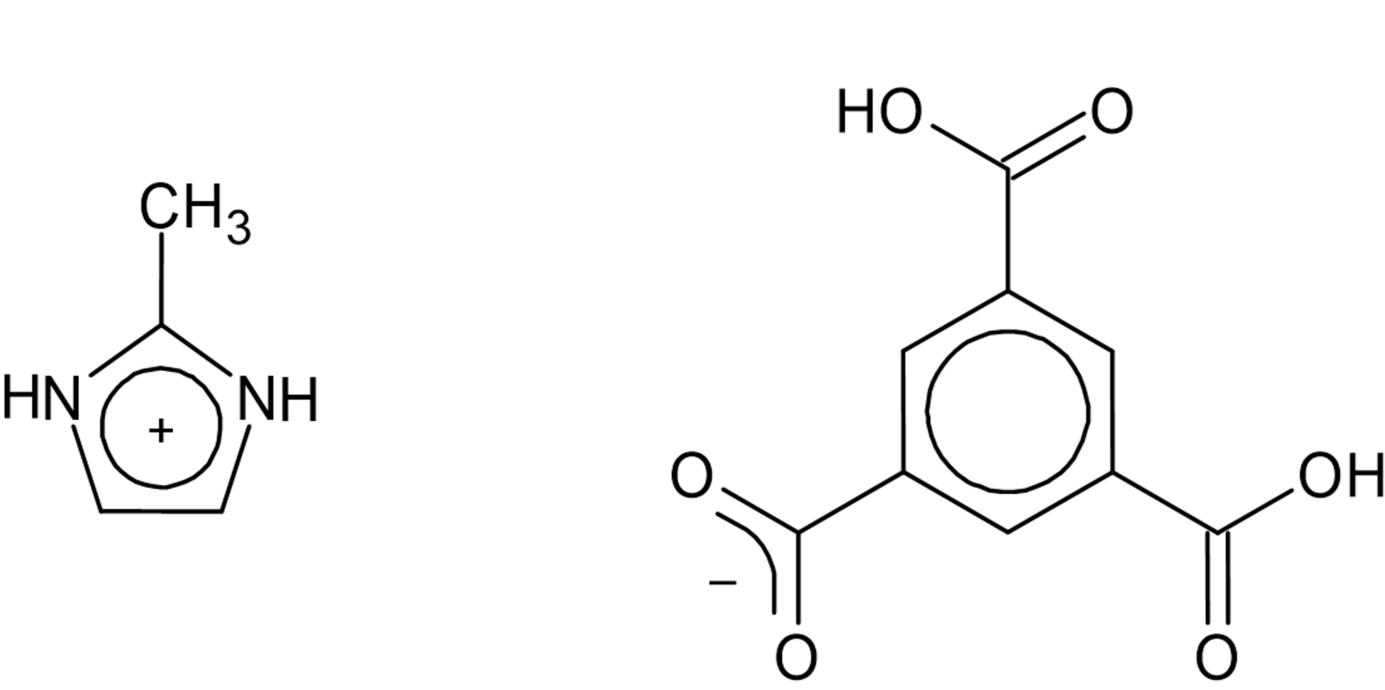


In a previous publication, we reported the complex hexa­aqua­cobalt bis­(2-methyl-1*H*-imidazol-3-ium) tetra­aqua­bis­(benzene-1,3,5-tri­carboxyl­ato-κ*O*)cobalt (**2**), synthesized at ambient conditions (Velazquez-Garcia & Techert, 2022 ▸). That work led us to modify the synthesis of the complex, resulting in the unexpected synthesis of the title compound.

## 2. Structural commentary

**Figure 1:**
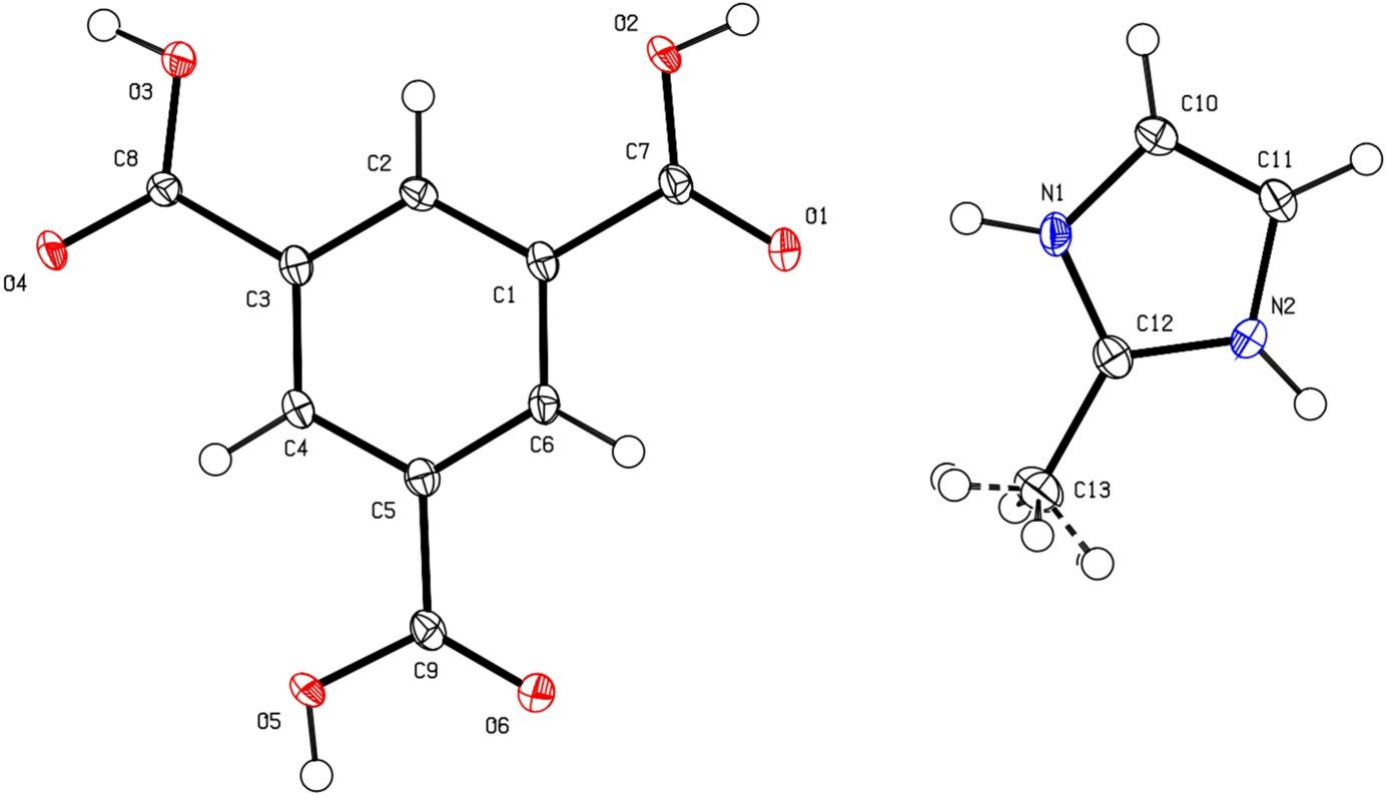
The mol­ecular structure of **1** with displacement ellipsoids drawn at the 50% probability level.

Compound **1** crystallizes with one singly deprotonated trimesate (btc) mol­ecule and one 2-methyl-1*H*-imidazol-3-ium (HmIm) mol­ecule in the asymmetric unit, space group *C*2/*c*. An ellipsoid plot illustrating these mol­ecules can be seen in Fig. 1 ▸. The hydrogen atoms attached to O2 and O3 lie in close vicinity to an inversion center or twofold axis, respectively, and as a consequence, each is disordered between two neighboring mol­ecules with equal occupancy.

Table 1 ▸ exhibits selected bond distances and angles of the btc ion. Among these bonds, the shortest non-hydrogen bond occurs between C9 and O6 with 1.224 (2) Å, while the largest is between C1 and C7 with 1.511 (2) Å. The O—C and C—C bond lengths are in the ranges 1.224 (2)–1.320 (2) Å and 1.388 (2)–1.511 (2) Å, respectively. These distances are slightly larger than those corresponding to the reported Hbtc mol­ecule (Tothadi *et al.*, 2020 ▸), which range between 1.229 (5) and 1.303 (5) Å for the O—C bond distances and between 1.381 (6) and 1.494 (9) Å for the C—C bond distances. Additionally, Hbtc exhibits O—C—O angles of the carboxyl­ate group ranging from 124.4 (4) to 125.0 (4)° and C—C—C angles of the aromatic ring ranging from 119.0 (4) to 121.1 (4)°, while btc shows slightly wider ranges with O—C—O falling in the 123.9 (2)–126.1 (2)° range and C—C—C angles in the 118.9 (2)–121.4 (4)° range.

**Figure 2:**
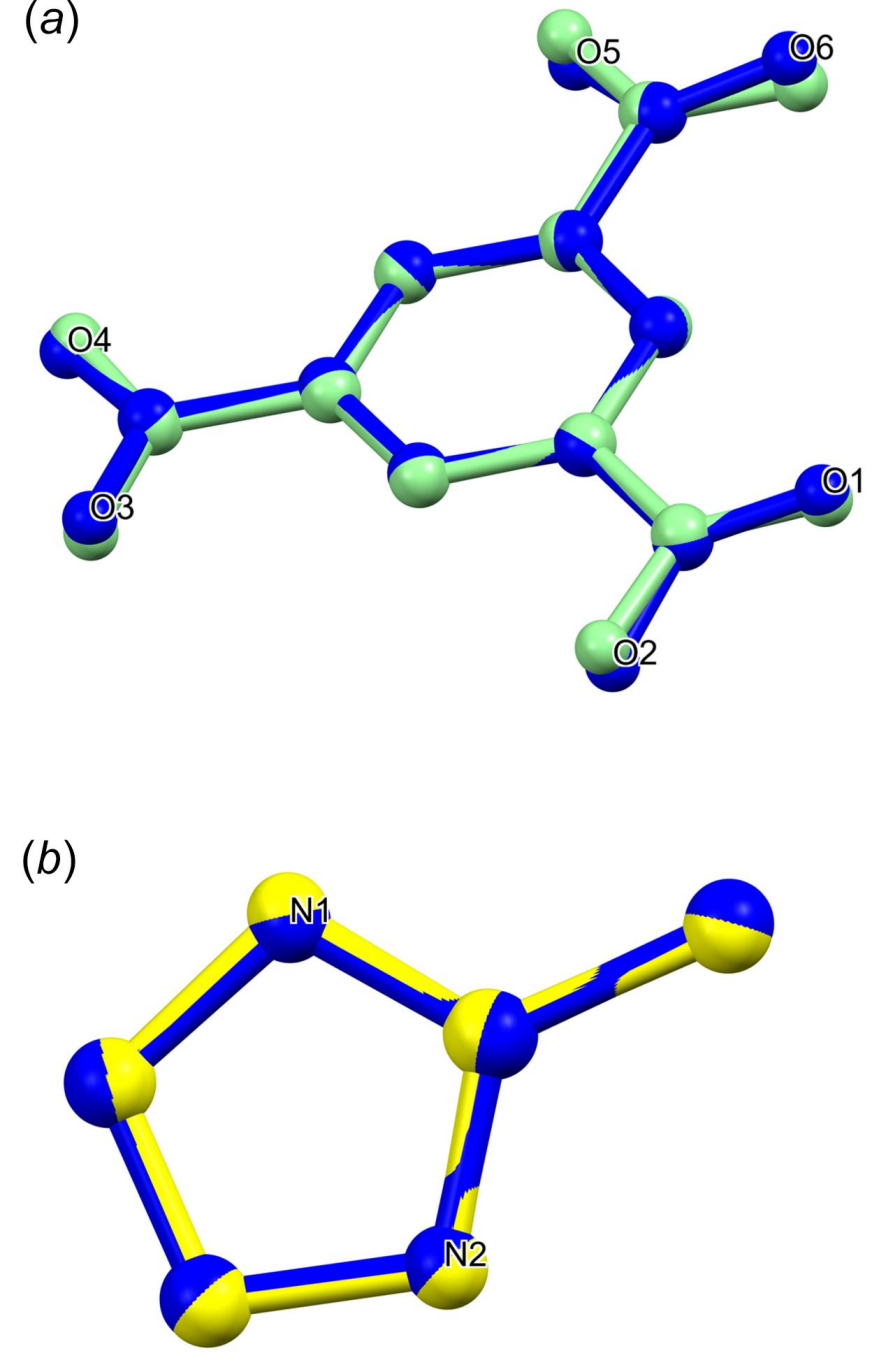
Mol­ecular overlay plot comparing (*a*) the btc ion in **1** (blue) *versus* the Hbtc mol­ecule (green; Tothadi *et al.*, 2020 ▸), and (*b*) the HmIm ion in **1** (blue) *versus* the mIm mol­ecule (yellow; Hachuła *et al.*, 2010 ▸)

The main difference between the Hbtc mol­ecule of Tothadi and co-workers and the btc ion within the present compound lies in their torsion angles. In the Hbtc mol­ecule, the oxygen atoms are nearly coplanar with the aromatic ring, with torsion angles deviating from 0 or 180° by no more than 4.2 (4)°. In contrast, the btc ion in compound **1** shows a wider deviation range, spanning from 4.2 (2) to 16.6 (2)°. Oxygen atoms O3 and O4 in **1** are the most coplanar with the aromatic ring, as illustrated by the torsion angles O3—C8—C3—C2 and O4—C8—C3—C4 of 4.4 (2) and 5.7 (3)°, respectively. The difference between Hbtc and btc is further highlighted through a mol­ecular overlay (Fig. 2 ▸) generated by the *Mercury* software (Macrae *et al.*, 2020 ▸). The root-mean-squared deviation (r.m.s.d.), as calculated by *Mercury* is 0.1356 Å, with the major distinction being in the positions of atoms O5 and O6 (Fig. 2 ▸ *a*).

Selected bond distances and angles for the mIm ion are presented in Table 2 ▸. The C—C bond distances are 1.345 (3) and 1.481 (3) Å, whereas the N—C distances range from 1.327 (2) to 1.377 (2) Å. These distances are slightly shorter than those found in the neutral mIm mol­ecule reported by Hachuła *et al.* (2010 ▸), where the C—C bond distances are 1.367 (1) and 1.488 (1) Å, and the N—C distances range from 1.329 (1) to 1.385 (1) Å. It is worth noting that imidazole derivatives often exhibit an asymmetry in the two endocyclic N—C bonds (Hachuła *et al.*, 2010 ▸), a characteristic also observed in compound **1**, where N1—C12 [1.326 (2) Å] shows greater double-bond character than N2—C12 [1.335 (2) Å]. However, this difference is more pronounced in the neutral mol­ecule [0.022 (1) Å] compared with the HmIm ion in **1** [0.008 (3) Å], possibly due to the protonation in the HmIm ion.

Compared with the neutral mIm mol­ecule, protonation in the HmIm ion results in a more symmetrical heterocyclic ring. This increase in the symmetry is observed in the C—C—N and N—C—N angles of the heterocyclic ring, which closely approach the ideal penta­gon angle of 108° in the HmIm ion, with a maximum deviation of 1.6 (2)°, while in the neutral mIm mol­ecule, this deviation is slightly larger, at 3.4 (1)°. However, in both cases the carbon of the methyl group is almost coplanar with the heterocycle ring as observed in the torsion angles C10—N1—C12—C13 and C11—N2—C12—C13 of −179.5 (2) and 179.6 (2)° for HmIm and −179.4 (1) and 179.3 (1) for mIm.

Fig. 2 ▸ *b* illustrates the mol­ecular overlay between the HmIm ion in compound **1** and the neutral mIm mol­ecule as reported by Hachuła and co-workers*.* The figure demonstrates that contrary to the btc ion, the HmIm ion bears a closer resemblance to its neutral counterpart. This similarity is further supported by the r.m.s.d. value calculated by *Mercury*, which has a value of 0.0320 Å.

## 3. Supra­molecular features

**Figure 3:**
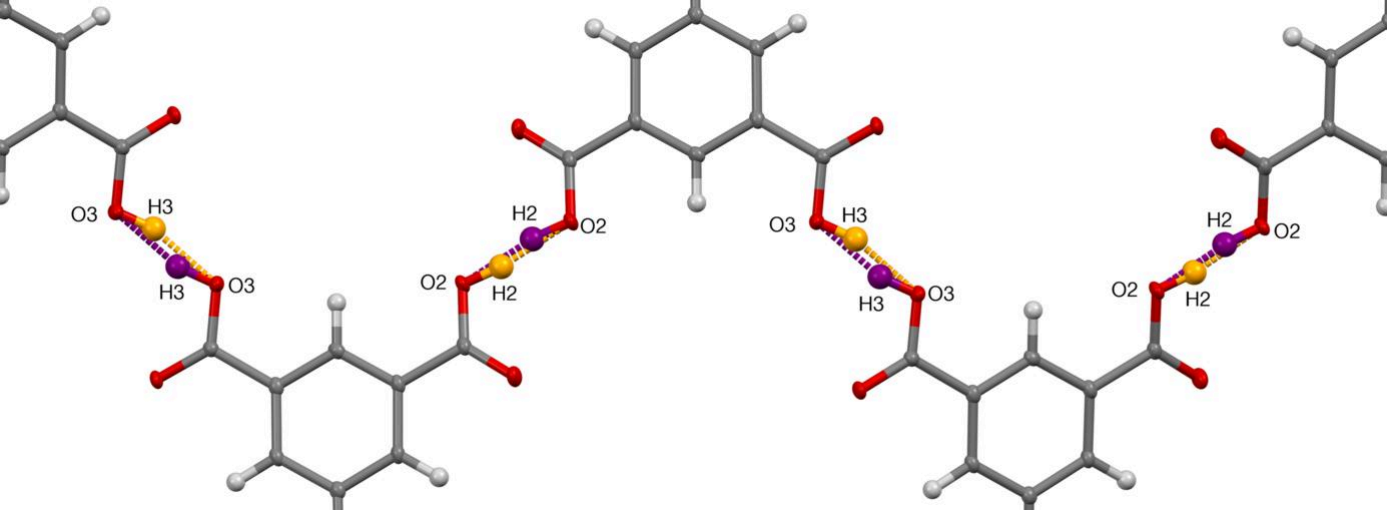
The two possible mutual positions of the hydrogen atoms H2 and H3 (orange or violet) and the resulting hydrogen bonds in the infinite chain of the trimesate anions.

**Figure 4:**
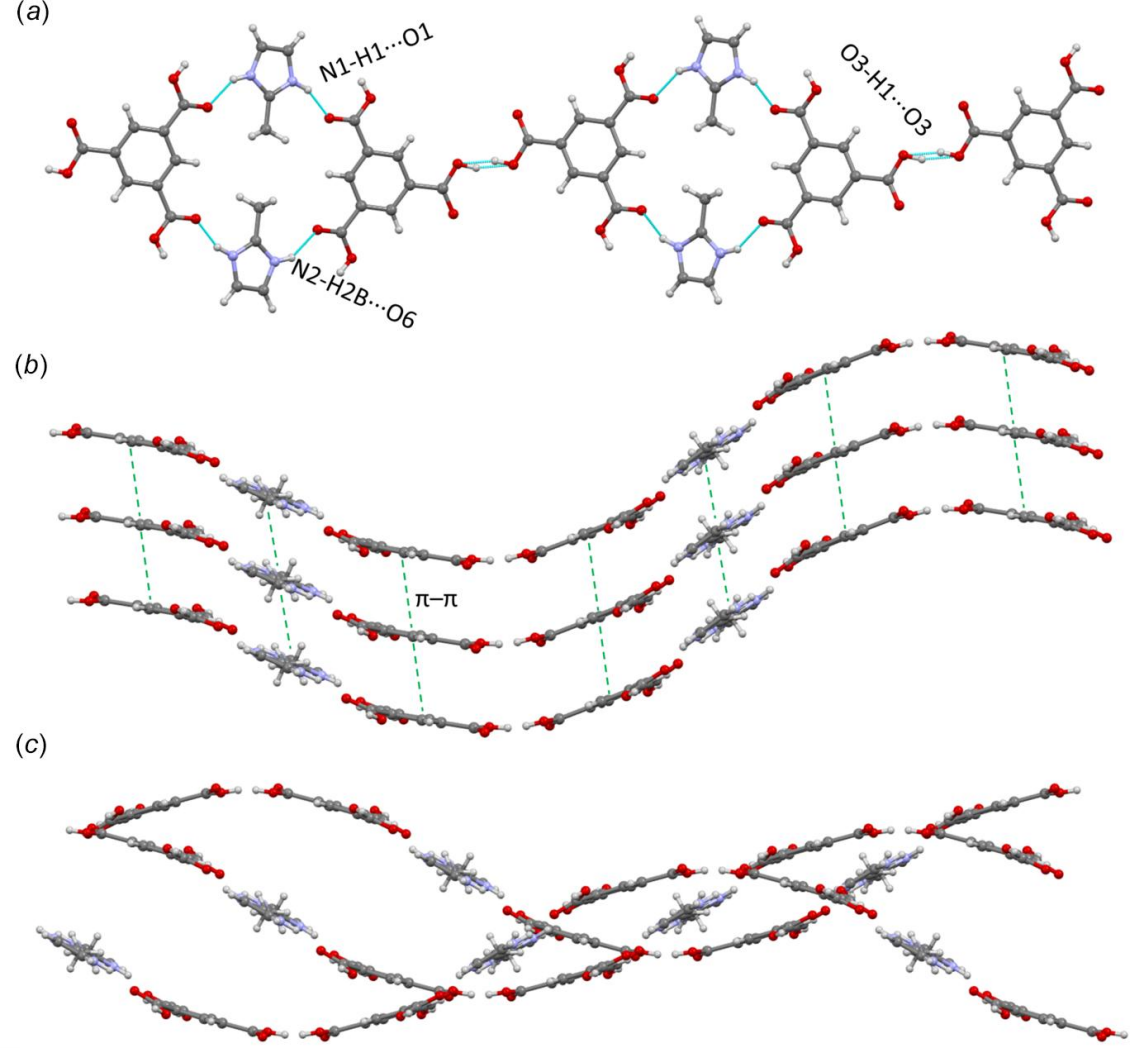
Crystal packing in compound **1.** (*a*) View down the *b* axis showing undulating chains formed by HmIm and btc ions through hydrogen bonding (blue lines), (*b*) view along the [101] direction illustrating the stacking of the chains *via* π–π inter­actions (green lines), and (*c*) view of the inter­connection of chains in an out-of-phase arrangement.

The crystal packing in **1** is primarily based on hydrogen bonds and π–π inter­actions. Table 3 ▸ provides a summary of the hydrogen bonds found within the compound. Hydrogen atoms H2 and H3 are involved in an infinite chain of hydrogen bonds. As a result of the symmetry of the crystal, and the negative charge of the trimesate anion, the protons H2 and H3 have an occupancy of only 50%, meaning that in the asymmetric unit, the negative charge is distributed evenly between the two carboxyl­ates. In other words, if O3 is protonated, O2 from the same mol­ecule is not and the neighboring trimesate mol­ecules participating in the hydrogen-bonded chain will have O2 protonated and O3 not (Fig. 3 ▸). As illustrated in Fig. 4 ▸ *a*, hydrogen bonds N1—H1⋯O1, N2—H2*B*⋯O6, and O3—H1⋯O3 form undulating chains that extend along the [ 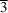 0 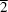 ] direction, while π–π inter­actions [centroid–centroid distance of 3.770 (2) Å], both among mIm and between btc ions, stack the chains along the *b*-axis direction (Fig. 4 ▸ *b*). Finally, hydrogen bonds O5—H5⋯O4 and O2—H2⋯O2 inter­connect the chains in an out-of-phase manner (Fig. 4 ▸ *c*), expanding the structure throughout the *ac* plane.

## 4. Database survey

A search for the title compound in the Cambridge Structural Database (CSD, Version 5.43, update of November 2022; Groom *et al.*, 2016 ▸) did not match with any reported structures. The structure of the neutral mIm mol­ecule has been reported with refcode FULPIM (Hachuła *et al.*, 2010 ▸), while several structures of Hbtc have been reported with refcodes BTCOAC01 (Duchamp & Marsh, 1969 ▸), BTCOAC03, FONHEW01, SOWCUF, SOWDIU, SOWDUG, SOWFAO, SOWFIW, SOWFOC (Cui *et al.*, 2019 ▸), BTCOAC05 (Tothadi *et al.*, 2020 ▸), CAFVOW, CAFVUC (Rajput *et al.*, 2010 ▸), FONHEW (Fan *et al.*, 2005 ▸), IYUQIC, IYUQOI (Dale *et al.*, 2004 ▸), LERSAD (Vishweshwar *et al.*, 2006 ▸), LUWWEI, LUWWEI01 (Yan *et al.*, 2020 ▸), MIMXEO, MIMXIS, MIMXOY, MIMXUE (Sanchez-Sala *et al.*, 2018 ▸), MIXCOM (Rodríguez-Cuamatzi *et al.*, 2007 ▸), OLAJIX01 (Ward & Oswald, 2020 ▸), QEYFIK (Goldberg & Bernstein, 2007 ▸), TMADMS (Herbstein *et al.*, 1978 ▸), TMADMS01 (Bernès *et al.*, 2008 ▸), TMADMS02, XASFAA01 (Li *et al.*, 2018 ▸), TRIMES10 (Herbstein & Marsh, 1977 ▸), TUBBAT (Melendez *et al.*, 1996 ▸), UDUMUC (Chen *et al.*, 2007 ▸), XASFAA01 (Davey *et al.*, 2013 ▸), XAVPOZ, XAVQEQ (Chatterjee *et al.*, 2000 ▸) and XAVPOZ01 (Dale & Elsegood, 2003 ▸). Other organic compounds with a low degree of similarity to the title compound were also found, for example refcodes: ILELAO (Li & Li, 2016 ▸), INACOQ (Li *et al.*, 2010 ▸), LUBHEX, LUBHIB, LUBHOH, LUBHUN, LUBJAV (Singh *et al.*, 2015 ▸), NUHBAU (Du *et al.*, 2009 ▸), RUDRAJ, RUDREN, RUDRIR (Akutagawa *et al.*, 1996 ▸), RUDRAJ and RUDREN (Herbstein *et al.*, 2002 ▸). However, these organic compounds do not contain either trimesic acid or 2-methyl­imidazole or their respective ions.

## 5. Synthesis and crystallization

In a typical synthesis, solutions of CoCl_2_·6H_2_O (2.5 ml, 0.02 *M*), mIm (65 µl, 1.58 *M*) and btc (500 µl, 0.12 *M*) were mixed without stirring. Within less than a minute, a blue precipitate was formed. The resulting heterogeneous mixture was allowed to slowly air-dry. After complete solvent evaporation, we obtained a mixture of the title compound, the previously reported cobalt complex **2**, and an unidentified phase. Although the blocky colorless crystals of the title compound can be easily identified in the mixture, all attempts to separate them from the other components by other than mechanical means were unsuccessful.

## 6. Refinement

Crystal data, data collection and structure refinement details are summarized in Table 4 ▸. The positions of hydrogen atoms were refined with *U*_iso_(H) = 1.2*U*_eq_(C or N) for CH and NH groups and *U*_iso_(H) = 1.5*U*_eq_(C or O) for others. Hydrogen atoms H2 and H3, each lying close to a symmetry element, were refined with a fixed occupancy of 0.5. The protons of the methyl group were refined as disordered over two geometrically idealized positions. The most disagreeable reflection (002) with an error/s.u. of more than 10 was omitted using the OMIT instruction in *SHELXL* (Sheldrick, 2015*b* ▸).

## Supplementary Material

Crystal structure: contains datablock(s) I. DOI: 10.1107/S2056989023009209/jq2031sup1.cif

Structure factors: contains datablock(s) I. DOI: 10.1107/S2056989023009209/jq2031Isup2.hkl

Supporting information file. DOI: 10.1107/S2056989023009209/jq2031Isup3.cml

CCDC reference: 2302266

Additional supporting information:  crystallographic information; 3D view; checkCIF report

## Figures and Tables

**Table 1:** Selected bond lengths (Å), bond angles (°), and torsion angles (°) of the btc ion

C1—C6	1.388 (2)	C2—C3—C4	118.91 (16)
C1—C7	1.511 (2)	C2—C1—C6	119.22 (16)
C2—C1	1.391 (3)	C5—C4—C3	120.01 (16)
C2—C3	1.393 (2)	C1—C6—C5	120.09 (16)
C4—C5	1.393 (3)	C4—C5—C6	120.36 (16)
C4—C3	1.396 (2)	C1—C2—C3	121.37 (17)
C6—C5	1.392 (2)	C2—C1—C7—O1	−168.16 (17)
C8—C3	1.505 (3)	C2—C1—C7—O2	12.3 (3)
C9—C5	1.485 (2)	C6—C1—C7—O1	10.5 (2)
O1—C7	1.236 (2)	C6—C1—C7—O2	−169.07 (16)
O2—C7	1.279 (2)	O3—C8—C3—C2	4.4 (2)
O3—C8	1.277 (2)	O3—C8—C3—C4	−174.16 (17)
O4—C8	1.247 (2)	O4—C8—C3—C2	−175.82 (17)
O5—C9	1.320 (2)	O4—C8—C3—C4	5.7 (3)
O6—C9	1.224 (2)	O5—C9—C5—C4	−16.1 (2)
O1—C7—O2	126.09 (16)	O5—C9—C5—C6	165.92 (15)
O4—C8—O3	124.26 (15)	O6—C9—C5—C4	163.43 (17)
O5—C9—O6	123.87 (16)	O6—C9—C5—C6	−14.5 (3)

**Table 2:** Selected bond lengths (Å), bond angles (°), and torsion angles (°) of the HmIm ion

C10—C11	1.345 (3)	N1—C12—C13	125.88 (17)
C12—C13	1.481 (3)	N2—C11—C10	106.37 (16)
N1—C12	1.327 (2)	N2—C12—C13	126.86 (17)
N1—C10	1.372 (2)	C12—N2—C11—C10	−0.0 (2)
N2—C12	1.335 (2)	C12—N1—C10—C11	−0.1 (2)
N2—C11	1.377 (2)	C10—N1—C12—C13	−179.50 (18)
C12—N1—C10	109.41 (15)	C11—N2—C12—C13	179.56 (18)
C12—N2—C11	109.55 (15)	C11—N2—C12—N1	−0.1 (2)
N1—C12—N2	107.26 (15)	C10—N1—C12—N2	0.1 (2)
N1—C10—C11	107.41 (16)	N1—C10—C11—N2	0.1 (2)

**Table 3:** Hydrogen-bond geometry (Å, °)

	D–H	H⋯A	D⋯A	D–H⋯A
N1–H1⋯O1	0.88	1.84	2.6771 (19)	159
O2–H2⋯O2^i^	0.84	1.64	2.4718 (16)	171
N2–H2*B*⋯O6^iv^	0.88	1.95	2.7460 (20)	151
O3–H3⋯O3^ii^	0.84	1.66	2.4601 (16)	159
O5–H5⋯O4^iii^	0.84	1.75	2.5840 (18)	170

(i) 1 − *x*,2 − *y*,1 − *z*, (ii) 1 − *x*,*y*,1/2 − *z*, (iii) 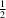 − *x*,- 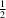 + *y*,1/2 − *z*, (iv) 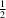 − *x*,1/2 − *y*,1 − *z*

**Table 4:** Experimental details

Crystal data	
Chemical formula	C_4_H_7_N_2_^+^·C_9_H_5_O_6_^−^
*M*_r_	292.25
Crystal system, space group	Monoclinic, *C*2/*c*
Temperature (K)	100
*a*, *b*, *c* (Å)	24.0655 (18), 3.7704 (3), 27.4258 (19)
β (°)	99.481 (8)
*V* (Å^3^)	2454.5 (3)
*Z*	8
Radiation type	Mo *K*α
μ (mm^−1^)	0.13
Crystal size (mm)	0.1 × 0.1 × 0.03

Data collection	
Diffractometer	Bruker APEX Duo CCD area detector
Absorption correction	Multi-scan (*SADABS*; Krause *et al.*, 2015 ▸)
*T*_min_, *T*_max_	0.628, 0.745
No. of measured, independent and observed [*I* > 2σ(*I*)] reflections	23840, 2531, 1969
*R*_int_	0.074
(sin θ/λ)_max_ (Å^−1^)	0.626

Refinement	
*R*[*F* ^2^ > 2σ(*F* ^2^)], *wR*(*F* ^2^), *S*	0.039, 0.103, 1.04
No. of reflections	2531
No. of parameters	204
No. of restraints	3
H-atom treatment	H atoms treated by a mixture of independent and constrained refinement
Δρ_max_, Δρ_min_ (e Å^−3^)	0.24, −0.28

Computer programs: *APEX2* and *SAINT* (Bruker, 2016 ▸), *SHELXT2018/2* (Sheldrick, 2015*a* ▸), *SHELXL2018/3* (Sheldrick, 2015*b* ▸), and *OLEX2* (Dolomanov *et al.*, 2009 ▸).

## supplementary crystallographic information

### Crystal data

**Table:**

C_4_H_7_N_2_^+^·C_9_H_5_O_6_^−^	*F*(000) = 1216
*M**_r_* = 292.25	*D*_x_ = 1.582 Mg m^−^^3^
Monoclinic, *C*2/*c*	Mo *K*α radiation, λ = 0.71073 Å
*a* = 24.0655 (18) Å	Cell parameters from 2955 reflections
*b* = 3.7704 (3) Å	θ = 2.5–30.4°
*c* = 27.4258 (19) Å	µ = 0.13 mm^−^^1^
β = 99.481 (8)°	*T* = 100 K
*V* = 2454.5 (3) Å^3^	Block, clear light colourless
*Z* = 8	0.1 × 0.1 × 0.03 mm

### Data collection

**Table:**

Bruker APEX Duo CCD area detector diffractometer	1969 reflections with *I* > 2σ(*I*)
Graphite monochromator	*R*_int_ = 0.074
phi and ω scans	θ_max_ = 26.4°, θ_min_ = 2.1°
Absorption correction: multi-scan (SADABS; Krause *et al.*, 2015)	*h* = −30→30
*T*_min_ = 0.628, *T*_max_ = 0.745	*k* = −4→4
23840 measured reflections	*l* = −34→34
2531 independent reflections	

### Refinement

**Table:**

Refinement on *F*^2^	Primary atom site location: dual
Least-squares matrix: full	Hydrogen site location: mixed
*R*[*F*^2^ > 2σ(*F*^2^)] = 0.039	H atoms treated by a mixture of independent and constrained refinement
*wR*(*F*^2^) = 0.103	*w* = 1/[σ^2^(*F*_o_^2^) + (0.0457*P*)^2^ + 3.154*P*] where *P* = (*F*_o_^2^ + 2*F*_c_^2^)/3
*S* = 1.04	(Δ/σ)_max_ < 0.001
2531 reflections	Δρ_max_ = 0.24 e Å^−^^3^
204 parameters	Δρ_min_ = −0.28 e Å^−^^3^
3 restraints	

### Special details

**Table:**

Geometry. All esds (except the esd in the dihedral angle between two l.s. planes) are estimated using the full covariance matrix. The cell esds are taken into account individually in the estimation of esds in distances, angles and torsion angles; correlations between esds in cell parameters are only used when they are defined by crystal symmetry. An approximate (isotropic) treatment of cell esds is used for estimating esds involving l.s. planes.

### Fractional atomic coordinates and isotropic or equivalent isotropic displacement parameters (Å^2^)

**Table:**

	*x*	*y*	*z*	*U*_iso_*/*U*_eq_	Occ. (<1)
O5	0.21243 (5)	1.0194 (4)	0.29386 (4)	0.0167 (3)	
H5	0.178804	0.950516	0.290143	0.025*	
O4	0.39014 (5)	1.2973 (4)	0.22895 (4)	0.0172 (3)	
O3	0.47026 (5)	1.2416 (4)	0.28243 (4)	0.0175 (3)	
H3	0.484491	1.273555	0.256792	0.026*	0.5
O1	0.39873 (5)	0.7849 (4)	0.48212 (4)	0.0196 (3)	
O2	0.47345 (5)	1.0439 (4)	0.45787 (4)	0.0174 (3)	
H2	0.488390	1.006191	0.487304	0.026*	0.5
O6	0.21810 (5)	0.7269 (4)	0.36581 (4)	0.0186 (3)	
N1	0.41365 (6)	0.4251 (4)	0.56743 (5)	0.0157 (3)	
H1	0.417821	0.532713	0.539790	0.019*	
N2	0.37701 (6)	0.1426 (4)	0.62208 (5)	0.0154 (3)	
H2B	0.352463	0.029800	0.636906	0.018*	
C2	0.41679 (7)	1.0776 (5)	0.36004 (6)	0.0127 (4)	
H2A	0.456452	1.111505	0.365596	0.015*	
C1	0.39023 (7)	0.9786 (5)	0.39933 (6)	0.0127 (4)	
C9	0.24019 (7)	0.8931 (5)	0.33578 (6)	0.0135 (4)	
C4	0.32831 (7)	1.0735 (5)	0.30481 (6)	0.0125 (4)	
H4	0.306933	1.108898	0.272846	0.015*	
C7	0.42279 (7)	0.9286 (5)	0.45087 (6)	0.0139 (4)	
C8	0.41665 (7)	1.2307 (5)	0.27097 (6)	0.0125 (4)	
C6	0.33258 (7)	0.9172 (5)	0.39079 (6)	0.0129 (4)	
H6	0.314219	0.841015	0.417075	0.016*	
C5	0.30162 (7)	0.9672 (5)	0.34375 (6)	0.0128 (4)	
C3	0.38646 (7)	1.1280 (5)	0.31275 (6)	0.0126 (4)	
C12	0.36643 (8)	0.2760 (5)	0.57639 (6)	0.0156 (4)	
C10	0.45497 (8)	0.3855 (5)	0.60797 (6)	0.0162 (4)	
H10	0.492702	0.467759	0.611105	0.019*	
C11	0.43223 (7)	0.2087 (5)	0.64244 (7)	0.0161 (4)	
H11	0.450624	0.142482	0.674491	0.019*	
C13	0.31239 (8)	0.2603 (6)	0.54168 (8)	0.0238 (4)	
H13A	0.308320	0.473526	0.520952	0.036*	0.47 (2)
H13B	0.281126	0.247390	0.560431	0.036*	0.47 (2)
H13C	0.311958	0.049737	0.520678	0.036*	0.47 (2)
H13D	0.2871 (17)	0.450 (11)	0.5494 (18)	0.036*	0.53 (2)
H13E	0.3175 (18)	0.267 (16)	0.5074 (8)	0.036*	0.53 (2)
H13F	0.2873 (16)	0.073 (11)	0.5495 (18)	0.036*	0.53 (2)

### Atomic displacement parameters (Å^2^)

**Table:**

	*U*^11^	*U*^22^	*U*^33^	*U*^12^	*U*^13^	*U*^23^
O5	0.0115 (6)	0.0275 (7)	0.0096 (6)	−0.0027 (5)	−0.0024 (5)	0.0013 (5)
O4	0.0140 (6)	0.0295 (8)	0.0073 (6)	0.0010 (5)	−0.0001 (5)	0.0032 (5)
O3	0.0119 (6)	0.0305 (8)	0.0101 (6)	−0.0004 (5)	0.0024 (5)	0.0024 (6)
O1	0.0187 (7)	0.0292 (8)	0.0105 (6)	−0.0031 (6)	0.0013 (5)	0.0052 (6)
O2	0.0132 (6)	0.0293 (8)	0.0085 (6)	−0.0020 (5)	−0.0020 (5)	0.0017 (6)
O6	0.0159 (6)	0.0265 (8)	0.0133 (6)	−0.0046 (6)	0.0019 (5)	0.0027 (6)
N1	0.0192 (8)	0.0185 (8)	0.0093 (7)	−0.0011 (6)	0.0024 (6)	0.0015 (6)
N2	0.0154 (7)	0.0171 (8)	0.0142 (7)	−0.0015 (6)	0.0043 (6)	0.0024 (6)
C2	0.0115 (8)	0.0138 (9)	0.0120 (8)	−0.0002 (7)	−0.0002 (7)	−0.0018 (7)
C1	0.0154 (8)	0.0134 (9)	0.0087 (8)	0.0003 (7)	0.0001 (7)	−0.0004 (7)
C9	0.0159 (9)	0.0153 (9)	0.0088 (8)	0.0006 (7)	0.0006 (7)	−0.0035 (7)
C4	0.0143 (8)	0.0133 (9)	0.0092 (8)	0.0008 (7)	−0.0004 (6)	0.0003 (7)
C7	0.0147 (8)	0.0170 (9)	0.0095 (8)	0.0023 (7)	0.0011 (7)	−0.0001 (7)
C8	0.0114 (8)	0.0152 (9)	0.0108 (8)	0.0000 (7)	0.0013 (6)	−0.0004 (7)
C6	0.0161 (9)	0.0139 (9)	0.0087 (8)	−0.0005 (7)	0.0018 (7)	0.0003 (7)
C5	0.0145 (8)	0.0129 (9)	0.0108 (8)	−0.0002 (7)	0.0014 (7)	−0.0015 (7)
C3	0.0155 (9)	0.0118 (9)	0.0101 (8)	0.0009 (7)	0.0013 (7)	−0.0004 (7)
C12	0.0176 (9)	0.0152 (9)	0.0136 (9)	0.0015 (7)	0.0009 (7)	−0.0013 (7)
C10	0.0152 (9)	0.0172 (9)	0.0156 (9)	−0.0003 (7)	0.0002 (7)	−0.0017 (7)
C11	0.0170 (9)	0.0181 (9)	0.0121 (9)	0.0029 (7)	−0.0002 (7)	0.0000 (7)
C13	0.0201 (10)	0.0277 (11)	0.0213 (10)	−0.0001 (8)	−0.0036 (8)	0.0003 (9)

### Geometric parameters (Å, º)

**Table:**

O5—H5	0.8400	C1—C6	1.388 (2)
O5—C9	1.320 (2)	C9—C5	1.485 (2)
O4—C8	1.247 (2)	C4—H4	0.9500
O3—H3	0.8400	C4—C5	1.393 (2)
O3—C8	1.277 (2)	C4—C3	1.396 (2)
O1—C7	1.235 (2)	C8—C3	1.505 (2)
O2—H2	0.8400	C6—H6	0.9500
O2—C7	1.279 (2)	C6—C5	1.392 (2)
O6—C9	1.224 (2)	C12—C13	1.481 (3)
N1—H1	0.8800	C10—H10	0.9500
N1—C12	1.326 (2)	C10—C11	1.345 (3)
N1—C10	1.372 (2)	C11—H11	0.9500
N2—H2B	0.8800	C13—H13A	0.9800
N2—C12	1.335 (2)	C13—H13B	0.9800
N2—C11	1.377 (2)	C13—H13C	0.9800
C2—H2A	0.9500	C13—H13D	0.984 (19)
C2—C1	1.391 (2)	C13—H13E	0.968 (19)
C2—C3	1.393 (2)	C13—H13F	0.976 (19)
C1—C7	1.511 (2)		
			
C9—O5—H5	109.5	C5—C6—H6	120.0
C8—O3—H3	109.5	C4—C5—C9	121.01 (15)
C7—O2—H2	109.5	C6—C5—C9	118.60 (15)
C12—N1—H1	125.3	C6—C5—C4	120.36 (16)
C12—N1—C10	109.41 (15)	C2—C3—C4	118.91 (16)
C10—N1—H1	125.3	C2—C3—C8	119.96 (15)
C12—N2—H2B	125.2	C4—C3—C8	121.11 (15)
C12—N2—C11	109.55 (15)	N1—C12—N2	107.26 (15)
C11—N2—H2B	125.2	N1—C12—C13	125.88 (17)
C1—C2—H2A	119.3	N2—C12—C13	126.86 (17)
C1—C2—C3	121.37 (16)	N1—C10—H10	126.3
C3—C2—H2A	119.3	C11—C10—N1	107.41 (16)
C2—C1—C7	121.63 (15)	C11—C10—H10	126.3
C6—C1—C2	119.22 (16)	N2—C11—H11	126.8
C6—C1—C7	119.14 (15)	C10—C11—N2	106.37 (16)
O5—C9—C5	114.15 (15)	C10—C11—H11	126.8
O6—C9—O5	123.87 (16)	C12—C13—H13A	109.5
O6—C9—C5	121.98 (15)	C12—C13—H13B	109.5
C5—C4—H4	120.0	C12—C13—H13C	109.5
C5—C4—C3	120.01 (16)	C12—C13—H13D	110 (3)
C3—C4—H4	120.0	C12—C13—H13E	113 (3)
O1—C7—O2	126.09 (16)	C12—C13—H13F	113 (3)
O1—C7—C1	118.28 (15)	H13A—C13—H13B	109.5
O2—C7—C1	115.64 (15)	H13A—C13—H13C	109.5
O4—C8—O3	124.26 (15)	H13B—C13—H13C	109.5
O4—C8—C3	121.17 (15)	H13D—C13—H13E	112 (4)
O3—C8—C3	114.57 (15)	H13D—C13—H13F	93 (4)
C1—C6—H6	120.0	H13E—C13—H13F	114 (4)
C1—C6—C5	120.09 (16)		
			
O5—C9—C5—C4	−16.1 (2)	C7—C1—C6—C5	178.97 (16)
O5—C9—C5—C6	165.92 (15)	C6—C1—C7—O1	10.5 (3)
O4—C8—C3—C2	−175.82 (17)	C6—C1—C7—O2	−169.07 (16)
O4—C8—C3—C4	5.7 (3)	C5—C4—C3—C2	−0.6 (3)
O3—C8—C3—C2	4.4 (2)	C5—C4—C3—C8	177.89 (16)
O3—C8—C3—C4	−174.11 (16)	C3—C2—C1—C7	−179.11 (16)
O6—C9—C5—C4	163.43 (17)	C3—C2—C1—C6	2.2 (3)
O6—C9—C5—C6	−14.5 (3)	C3—C4—C5—C9	−177.44 (16)
N1—C10—C11—N2	0.1 (2)	C3—C4—C5—C6	0.5 (3)
C2—C1—C7—O1	−168.16 (17)	C12—N1—C10—C11	−0.2 (2)
C2—C1—C7—O2	12.3 (3)	C12—N2—C11—C10	0.0 (2)
C2—C1—C6—C5	−2.3 (3)	C10—N1—C12—N2	0.1 (2)
C1—C2—C3—C4	−0.7 (3)	C10—N1—C12—C13	−179.50 (18)
C1—C2—C3—C8	−179.26 (16)	C11—N2—C12—N1	−0.1 (2)
C1—C6—C5—C9	178.97 (16)	C11—N2—C12—C13	179.56 (18)
C1—C6—C5—C4	1.0 (3)		

### Hydrogen-bond geometry (Å, º)

**Table:**

*D*—H···*A*	*D*—H	H···*A*	*D*···*A*	*D*—H···*A*
N1—H1···O1	0.88	1.84	2.6771 (19)	159
O2—H2···O2^i^	0.84	1.64	2.4718 (16)	171
N2—H2*B*···O6^ii^	0.88	1.95	2.746 (2)	151
O3—H3···O3^iii^	0.84	1.66	2.4601 (16)	159
O5—H5···O4^iv^	0.84	1.75	2.5840 (18)	170

Symmetry codes: (i) −*x*+1, −*y*+2, −*z*+1; (ii) −*x*+1/2, −*y*+1/2, −*z*+1; (iii) −*x*+1, *y*, −*z*+1/2; (iv) −*x*+1/2, *y*−1/2, −*z*+1/2.
